# Is Tourette syndrome a rare condition?

**DOI:** 10.12688/f1000research.53134.2

**Published:** 2021-11-02

**Authors:** Andreas Hartmann, Natalia Szejko, Nanette Mol Debes, Andrea E. Cavanna, Kirsten Müller-Vahl

**Affiliations:** 1 National Reference Center for Tourette Syndrome, Department of Neurology, Groupe Hospitalier Pitié-Salpêtrière, Paris, 75013, France; 2 Department of Neurology, Medical University of Warsaw, Warsaw, Poland; 3 Department of Bioethics, Medical University of Warsaw, Waraw, Poland; 4 Department of Pediatrics, Herlev University Hospital, Copenhagen, Denmark; 5 Department of Neuropsychiatry, BSMHFT and University of Birmingham, Birmingham, UK; 6 School of Life and Health Sciences, Aston University, Birmingham, UK; 7 Institute of Neurology, University College London, London, UK; 8 Clinic of Psychiatry, Socialpsychiatry and Psychotherapy, Hannover Medical School, Hannover, Germany

**Keywords:** Tourette syndrome, tics, rare disease, orphan disease

## Abstract

Based on its prevalence, Tourette syndrome cannot be considered a rare condition. However, in this opinion article, we make the claim that it should nonetheless be considered as an orphan or neglected disease.

## What is a rare disease?

Rare diseases are diseases which affect a small number of people compared to the general population. In Europe, a disease is considered to be rare when it affects 1 person per 2,000. ^1^ In contrast, the European Commission on Public Health ^2^ defines rare diseases not only based on low prevalence (<1 in 2,000 people), but in addition as “life-threatening or chronically debilitating diseases which are of such low prevalence that special combined efforts are needed to address them.” Accordingly, diseases that are statistically rare, but not also life-threatening, chronically debilitating, or inadequately treated, are excluded from this definition. In the United States, the Rare Diseases Act of 2002 ^3^ also defines rare disease strictly according to prevalence, but on the basis of different rates, as follows: “Any disease or condition that affects fewer than 200,000 people in the United States”, or about 1 in 1,500 people. ^4^

Because of definitions that include reference to treatment availability, a lack of resources, and severity of the disease, the term “orphan disease” has been introduced as a synonym for “rare disease”. Interestingly, in the United States and the European Union, orphan diseases have a distinct legal meaning. Originally, the orphan drug movement began in the United States. The United States Orphan Drug Act ^5^ summarizes under the term “orphan diseases” both rare diseases and any non-rare diseases “for which there is no reasonable expectation that the cost of developing and making available in the United States a drug for such disease or condition will be recovered from sales in the United States of such drug”. Similarly, the European Organization for Rare Diseases (EURORDIS ^6^) also includes both rare diseases and neglected diseases into a larger category of “orphan diseases”.

## Definition(s) of Tourette syndrome

Tourette syndrome (TS) is defined by the DSM-5 as a chronic tic disorder with the presence of at least two motor and one vocal tics over a period >12 months in someone under the age of 18 after excluding secondary causes (APA, 2013). This definition is not substantially different from that given by the previous version, the DSM-IV-TR, on which the current epidemiological literature is based. However, there was a major shift between the initial version of the DSM-IV and its revision: it was no longer a requirement that tics must be *debilitating.* This essentially descriptive vision of TS has its merits as it is difficult if not impossible to define operational criteria for what can be considered debilitating. Also, the waxing and waning nature of tics, both phenomenologically and with regard to severity, means that impairment may vary over time, even if the overall condition can be considered chronic. However, this very broad definition of TS also means that likely many people fall under this diagnostic umbrella category who do not at all require medical attention at any time during their life. The need to achieve a balance between the gains and losses resulting from the removal of the “impairment” criterion could be fruitfully reassessed a few decades after the change took place. The positive repercussions have been particularly manifest in the domain of research (e.g. inclusion of milder cases in genetic studies, cf. Müller-Vahl *et al.*, 2019). However, over time many clinicians working in specialist settings have noticed that a substantial degree of overlap between the definition of TS and what is observed in everyday clinical practice has inevitably been lost. In this respect, we acknowledge that there is an ongoing debate on whether to ground TS – among other neurodevelopmental conditions such as autism spectrum disorder (ASD) or attention deficit hyperactivity disorder (ADHD) – in the neurodiversity field. Which means, among other things, letting go of terms like “disease” or “disorder”, at least for a subset of concerned people, as these may be considered stigmatizing. For the same reason we do not encourage the use of terms like “handicap” or “disability” in relation to these conditions. We consider this debate to be important, but we endorse the premise that people with tics typically seek medical attention out of their own volition (there is no need to advertise our services and waiting lists are, unfortunately, much too long – see below). Therefore, we posit that terms such as “disease” of “disorder” are not entirely out of place, even though we fully acknowledge that impairment related to tics and/or comorbidities can fluctuate significantly, both inter- and intra-individually (see above). In consideration of such fleetingness, we are not in favour of reintroducing the ‘impairment’ criterion to the DSM – we simply point out that its removal has created problems of its own. However, in our opinion, there is no perfect solution to these dilemmas, just compromises. At the end of the day, classifications (DSM, ICD and others) and the way medical departments are structured are rather irrelevant when compared to the demands people in need – suffering people – place on us. Calling these people “patients” carries no stigma in our opinion: one wonders why any medical diagnosis should carry stigma, to begin with. Moreover, it can be argued that some bullying and harassment people with TS experience stems precisely from the fact that their tics are not recognized as a movement disorder; rather, they find themselves accused of “doing it on purpose”, of trying to attract attention, etc. Therefore, a fine line is being thread.

## Epidemiology of Tourette syndrome

Recent epidemiological studies of TS have estimated its prevalence between 0.3 to 0.7% in school-aged children ( Knight *et al*., 2012; Scharf *et al*., 2015). A conservative estimate would be around 0.5%, that is one child in 200, 10 times the accepted rate for a rare disease. In adults there is no solid epidemiological data although we may extrapolate for the pediatric findings. Assuming that two thirds of patients with TS remit when entering adulthood ( Leckman *et al*., 1998), around 0.2% of adults might still suffer from TS, which still does not fulfill any of the diagnostic criteria for rare diseases given above. A recent meta-analysis based on only three studies, however, suggested a prevalence rate of 0.012%, which would make adult TS indeed a rare disease ( Levine *et al*., 2019). Clearly, more epidemiological research is warranted in the field of adult TS.

## The problem

What experts on TS agree on is that the condition is underdiagnosed, and the delay between onset of symptoms (tics) and diagnosis is too long ( Mol Debes *et al.*, 2008; Shilon *et al*., 2008). Yet, they also acknowledge that a substantial number of people, regardless of age (but likely more adults) remain unbothered by their tics and live perfectly normal lives; in other words, they never seek medical attention and have no reason to do so. Based on this fact, the term “tic spectrum disorder” has been suggested including all variants and severity levels of the disease ( Müller-Vahl *et al.*, 2019).

However, in an ideal world, how many people with TS according to DSM-5 criteria might legitimately be called *patients*? We speculate the percentage to be around 10-20%. One of the strategies to tackle this problem could be division of patients with TS in clinical subgroups mainly dependent on tic severity. According to this premise, severe TS is indeed, and thankfully, a rare condition. But here also, tolerance to tics and their sequalae, both social and functional, differ enormously from patient to patient, so defining operational criteria or some sort of cut-off (i.e., on the Yale Global Tic Severity Scale) will most likely be impossible. A further strategy could be pursued by focusing on the complexity or phenotype – with and without comorbidity – of the clinical picture of TS. This strategy might involve a return to the origin, namely to Gilles de la Tourette’s original definition of TS, which encompasses the symptom triad of tics, echolalia, and coprolalia. The condition presenting with both simple and complex tics is sometimes referred to as “full-blown TS” and would arguably qualify for the “rare disease” category ( Cavanna, 2018). The abovementioned strategies would lead to the identification of a subgroup of patients whose condition bears a clinically relevant impact on quality of life. This pathway could compensate for the information lost with the removal of the “impairment” criterion from the revised set of diagnostic criteria for TS in the DSM-IV-TR (2000). Of note, there have also been efforts to define evidence-based TS subgroups through factor or cluster analyses, although these have not yet had a significant impact on diagnosis and management (cf. Cravedi et al., 2018). Finally, and in this respect, one of the most important tasks in TS research will be to gather more solid epidemiological and longitudinal data ( Black et al., 2021).

## A proposal

Although tics are a common symptom, we wish to officially continue considering TS a rare (or orphan) disease because of certain public health (and other) benefits this implies. Clearly, TS is underrecognized (or misrepresented – Fat *et al*., 2012) and underdiagnosed, so, *de facto*, rare for treating physicians and other health care professionals. There is very little targeted drug research for the treatment of tics to date, and in most patients off-label prescriptions are made. For these reasons, we might reasonably call TS a *neglected* disease, needing further support as a “rare disease” by policy makers until things change. In other words, TS as such might not be rare per se, but there clearly is a paucity of available and accessible treatments for this patient population. Those insisting on high prevalence of TS to increase support/awareness of this condition may, ironically, render a disservice to patients. Similar to patients with essential tremor, we will then be left to our own devices in the no man’s land between public and industrial support, and progress may slowly grind to a halt.

## Data availability

No data is associated with this article.

## Footnotes

^1^

https://en.wikipedia.org/wiki/Rare_disease.

^2^

https://en.wikipedia.org/wiki/European_Commission.

^3^

https://en.wikipedia.org/wiki/Rare_Diseases_Act_of_2002 .

^4^

https://rarediseases.info.nih.gov/diseases/pages/31/faqs-about-rare-diseases .

^5^

https://en.wikipedia.org/wiki/Orphan_Drug_Act .

^6^

www.eurordis.org.
