# Supplementary material for: Is Tourette syndrome a rare condition?
Source: F1000Res. 2021 Nov 2;10:434. Originally published 2021 Jun 2. [Version 2] doi: 10.12688/f1000research.53134.2 (PMC8567683; doi:10.12688/f1000research.53134.2)
Supplement: Peer review history

# Peer review: Reviewer response for version 2

**DOI:** 10.5256/f1000research.78188.r98694

**Published:** 2021-11-03

**Authors:** Peter Nagy

**Affiliations:** 1 Vadaskert Hospital and Outpatient, Budapest, Hungary

Thank for the responses, I have no further comment.

Is the topic of the opinion article discussed accurately in the context of the current literature?

Yes

Are arguments sufficiently supported by evidence from the published literature?

Yes

Are all factual statements correct and adequately supported by citations?

Yes

Are the conclusions drawn balanced and justified on the basis of the presented arguments?

Partly

Reviewer Expertise:

neurodevelopmental disorders, psychopharmacology

I confirm that I have read this submission and believe that I have an appropriate level of expertise to confirm that it is of an acceptable scientific standard.

# Peer review: Reviewer response for version 2

**DOI:** 10.5256/f1000research.78188.r98693

**Published:** 2021-11-03

**Authors:** Tammy Hedderly, Osman Malik, Tamsin Owen

**Affiliations:** 1 Tic and Neurodevelopmental Movements Service (TANDeM), Evelina Children's Hospital, London, UK; 2 Evelina London Children's Healthcare, Guy's and St Thomas's, London, UK; 3 TANDeM, Evelina London Children's Healthcare, Guys and St Thomas's NHS Trust, London, UK

Thank you to the authors for providing a clearer explanation to the proposals and for also highlighting important points for future debate in our field. We are happy to agree to the submissions and look forward to further discussions as always. TH

Is the topic of the opinion article discussed accurately in the context of the current literature?

Yes

Are arguments sufficiently supported by evidence from the published literature?

Yes

Are all factual statements correct and adequately supported by citations?

Yes

Are the conclusions drawn balanced and justified on the basis of the presented arguments?

Yes

Reviewer Expertise:

Paediatric Movement Disorders and Neurodevelopmental medicine

We confirm that we have read this submission and believe that we have an appropriate level of expertise to confirm that it is of an acceptable scientific standard.

# Peer review: Reviewer response for version 1

**DOI:** 10.5256/f1000research.56487.r87458

**Published:** 2021-07-16

**Authors:** Tammy Hedderly, Tamsin Owen, Osman Malik

**Affiliations:** 1 Tic and Neurodevelopmental Movements Service (TANDeM), Evelina Children's Hospital, London, UK; 2 TANDeM, Evelina London Children's Healthcare, Guys and St Thomas's NHS Trust, London, UK; 3 Evelina London Children's Healthcare, Guy's and St Thomas's, London, UK

This viewpoint article, with its interesting challenge to consider Tourette syndrome (TS) as a rare (or orphan) disease, has come during an unfortunate moment in time where we are experiencing a Covid associated pandemic of tic and tic-like-movements referrals (Heyman, Liang and Hedderly 2021). We are reflecting on these concepts whilst also sitting amongst an ever-increasing pile of new referrals for children and adolescents with a dramatic increase in tics and functional tic-like attacks, making the current prevalence rate far from ‘rare’.

 The viewpoint paper opens with a title that, in itself, raises controversy. Should TS even be considered a disease at all? When engaging with some of the children and families with tics we often embrace terms such as developmental differences and neuro diversity so as to reduce the stigma sometimes associated with tics. We often encourage children to re-frame their tics and co-morbidities as diversity and difference in order to focus on some of the benefits this neurodiversity brings (for example heightened attention to detail, perfectionist traits or special abilities to switch attention)We routinely do this when the situation allows accepting, of course, for some of the ‘patients’ that more detailed therapeutic strategies are needed to enable individuals to achieve a therapeutic goal.  Should the term disease be therefore removed from the discussion?

 The proposed postulate should probably remind us all of the lively (and fun) debate held at the European Society of Tourette Syndrome (ESSTS) in Copenhagen in 2018 (?) when we were all fortunate enough to meet face to face. At this meeting, we asked if ‘Syndrome’ within Tourette should be replaced with ‘spectrum’ instead, a discussion which helpfully involved those with lived experience of Tourette syndrome. To our unit’s surprise the vote was in favour of keeping the term syndrome, as we had personally changed to using the term Tic/Tourette Spectrum within our own clinical service in 2012. We published our rationale for this in a paediatric journal (Malik and Hedderly 2018 ) and our unit’s patient surveys revealed a preference for this term. We were pleased to see that our colleagues in Germany (Muller-Vahl 2019) support this preference. We feel this user-led preference for less pathologizing language supports not viewing Tourette syndrome as a disease. It would be interesting to hear the views of individuals with lived experience of Tourette syndrome about the palatability of the term “rare disease” to describe their symptoms.

 This viewpoint paper provides an engaging discussion around how definitions of rarity of disease and orphan status vary depending on which area the reader sits in, whether in UK, USA or in the European Commission of Public Health. An informative point is raised about the lack of operational criteria for applying the term ‘debilitating’ and the removal if this criteria in DSM -V. The authors also point out helpfully that when using the term ‘wax and wane’ the clinicians should remember this does not just apply to the tic phenomenology but also to how impairing the symptoms are at any given time, although personally we recommend avoiding the use of the term handicap. This is so that we can fully respect people presenting with difficulties or with disability as individuals who can still have control over their own lives.

 In this article, the term TS is highlighted to be used very much as an umbrella term including people with no presenting complaints and those who do not seek medical attention. The authors appear to advocate a re-evaluation of the term ‘impairment’ from the diagnostic criteria, whilst at the same time acknowledging that removing this term has had some positive benefits such as allowing for more cases to be included in genetic studies.

 The final position is not entirely clear and we would like to propose the need to consider the views of a user group-the children, adolescents and adults experiencing tics and symptoms or signs of Tourette Spectrum conditions. We would propose again the need to identify different clusters and subgroups of ‘Tourette experiencers’ and, although tics will be at the core of each cluster, we would propose that the defining features of different groups should not involve markers of tic severity at all moving away from the importance placed on using the ‘tic count’.

 The presence of the co-occuring conditions with a detailed formulation around driving forces to the tics are probably much more relevant (i.e. cluster groups). For example, in the novel currently exploding phenotype which (in our UK experience) has a higher representation of teenage girls with marked social anxiety and self-harm rates reaching 44%, we also see other groups with toe walking and autism traits and the emotionally dysregulated inattentive boys. The clinical cluster view involves making a patient-led, hierarchy of important ‘wishes’ to prioritise for potential modification.

 We propose that the social and functional difficulties described following tics may be better viewed as primary drivers of tics in some families and not just sequalae, as mentioned in the viewpoint paper.  We understand that potentially histaminergic, serotoninergic, dopaminergic, glutaminergic genes and pathways are involved in the generation of tics and tic-like behaviour and in the co-occuring mental health associations. The decision about how rare or not a Tic cluster may be will probably depend on the old dilemma of ‘lumping vs splitting’. Some of the newer bioinformatic approaches of research will ultimately answer the question of which of the pathogenetic mechanisms underpinning the Tourette Spectrum are rarer that the others.

 In conclusion, we would propose that Tourette syndrome should not be considered a rare disease due to the current prevalence rates, the potential impact on the user group and a need to consider the often frequent co-occurring symptoms. We prioritise an approach which includes the provision of a strength- and needs-based formulation. It is of course imperative to hold user-led consultation s regarding these discussions. In the meantime, we would propose not calling TS rare or an orphan ‘disorder or disease’ and instead highlight that a major problem in the field is the ‘rarity’ of available and accessible treatments. In the UK the CAMHS services are overloaded and our movement disorder clinics have long waiting lists to even get to the point of diagnosis. Therapists trained in the management of tics or functional tic like episodes are scarce. Working together we must aim to address the orphan-ness or rarity of the resources to help provide solutions to the users with ever-increasing problems that we are all seeing in our clinics.

**References:**

 Malik O, Hedderly T. Childhood tic disorders: diagnosis and management. Symposium: Neurology| Volume 28, Issue 10, P445-453, October 01, 2018

 Müller-Vahl KR, Sambrani T, Jakubovski E. Tic disorders revisited: introduction of the term "tic spectrum disorders". Eur Child Adolesc Psychiatry. 2019 Aug;28(8):1129-1135.

 Heyman I, Liang H, Hedderly T. COVID-19 related increase in childhood tics and tic-like attacks. Arch Dis Child. 2021 Mar 6:archdischild-2021-321748. doi: 10.1136/archdischild-2021-321748. Epub ahead of print. PMID: 33677431.

Is the topic of the opinion article discussed accurately in the context of the current literature?

Yes

Are arguments sufficiently supported by evidence from the published literature?

Yes

Are all factual statements correct and adequately supported by citations?

Yes

Are the conclusions drawn balanced and justified on the basis of the presented arguments?

Yes

Reviewer Expertise:

Paediatric Movement Disorders and Neurodevelopmental medicine

We confirm that we have read this submission and believe that we have an appropriate level of expertise to confirm that it is of an acceptable scientific standard, however we have significant reservations, as outlined above.

## References

[1] : COVID-19 related increase in childhood tics and tic-like attacks. *Arch Dis Child* .2021; 10.1136/archdischild-2021-321748 10.1136/archdischild-2021-321748 33677431

[2] : Childhood tic disorders: diagnosis and management. *Paediatrics and Child Health* .2018;28(10) : 10.1016/j.paed.2018.07.007 445-453 10.1016/j.paed.2018.07.007

[3] : Tic disorders revisited: introduction of the term "tic spectrum disorders". *Eur Child Adolesc Psychiatry* .2019;28(8) : 10.1007/s00787-018-01272-7 1129-1135 10.1007/s00787-018-01272-7 30661132PMC6675752

# Response

**Published:** 2021-07-26

**Authors:** Andreas Hartmann

**Affiliations:** Pitié-Salpêtrière Hospital, Paris, France

We thank Dr Hedderly for her thoughtful comments.

 To begin with, we acknowledge that there is an ongoing debate on whether to ground TS – among other neurodevelopmental conditions such as autism spectrum disorder (ASD) or attention deficit hyperactivity disorder (ADHD) – in the neurodiversity field. Which means, among other things, letting go of terms like “disease” or “disorder”, at least for a subset of concerned people, as these may be considered stigmatizing. And, as also mentioned by Dr Hedderly, refrain for using terms like “handicap” or “disability” in relation to these conditions. We consider this debate to be important, but it is not specifically the subject of our article as we depart from three premises: (i) that TS is listed in the DSM-5 and thus, by definition, a medical condition; and (ii) that various health care professionals are following people with tics; and (iii) most importantly – that people with tics seek medical attention out of their own volition (there is no need to advertise our services and waiting lists are, unfortunately, much too long, see below). Therefore, we posit that terms such as “disease” of “disorder” are not entirely out of place, even though we fully acknowledge that impairment related to tics and/or comorbidities can fluctuate significantly, both inter- and intra-individually, as also stated in our article. Because of that fleetingness, we are actually not in favour of reintroducing the ‘impairment’ criterion to the DSM – we simply point out that removing it has created problems of its own. However, in our opinion, there is no perfect solution to these dilemmas, just compromises. At the end of the day, classifications (DSM, ICD and others) and the way medical departments are constructed are rather irrelevant when compared to the demands people in need – *suffering* people – place on us. And, calling these people “patients” carries no stigma in our opinion: one wonders why any medical diagnosis should carry stigma, to begin with. Moreover, it can be argued that some bullying and harassment people with TS experience stems precisely from the fact that their tics are not recognized as a movement disorder; rather, they find themselves accused of “doing it on purpose”, of trying to attract attention, etc.

 As we may agree that some people may be incapacitated from tics and comorbidities, Dr Hedderly indeed points out that our departments are in high demand, with severe case loads and long waiting lists. And this is where our points of view merge: neither chronic tics nor TS are rare, numerically speaking (again, we simply conform to official definitions of rarity – 1:2000 prevalence rate – which can be debated in itself); but there are not enough trained experts, providers, and infrastructure around to adequately take care of these patients. Which, in effect, makes TS rare which is, for us, a matter-of-fact statement – but also a call for action. This unsatisfactory situation may be an opportunity to secure support and funding from regional, national or European bodies to help the TS community – both patients and healthcare providers. One example is COST action BM905 (2010-2014) which was instrumental in creating a European network on TS and gave a tremendous boost to the European Society for the Study of Tourette Syndrome (ESSTS); which, since its inception, has always entertained close ties to patient associations which have now organized in an international network (Tics and Tourette across the Globe - TTAG).

 So, to rephrase: TS is not rare but should be supported as such by public bodies as long as adequate research and medical care is not available in so many countries around the globe. Once TS has become more “mainstream” and is on the map of the general public, government agencies, but also the private sector, and across countries and continents, we’ll be happy to join the ranks of non-rare (common) diseases.

# Response

**Published:** 2021-10-04

**Authors:** Andreas Hartmann

**Affiliations:** Pitié-Salpêtrière Hospital, Paris, France

We thank Dr Hedderly for her thoughtful comments.

 To begin with, we acknowledge that there is an ongoing debate on whether to ground TS – among other neurodevelopmental conditions such as autism spectrum disorder (ASD) or attention deficit hyperactivity disorder (ADHD) – in the neurodiversity field. Which means, among other things, letting go of terms like “disease” or “disorder”, at least for a subset of concerned people, as these may be considered stigmatizing. For the same reason, as also mentioned by Dr Hedderly, we refrain from using terms like “handicap” or “disability” in relation to these conditions. We consider this debate to be important, but it is not specifically the subject of our article as we endorse three premises: (i) that TS is listed in the DSM-5 and thus, by definition, a medical condition; (ii) that various health care professionals are involved in the care of people with tics; and (iii) most importantly – that people with tics typically seek medical attention out of their own volition (there is no need to advertise our services and waiting lists are, unfortunately, much too long - see below). Therefore, we posit that terms such as “disease” of “disorder” are not entirely out of place, even though we fully acknowledge that impairment related to tics and/or comorbidities can fluctuate significantly, both inter- and intra-individually, as also stated in our article. In consideration of such fleetingness, we are actually not in favour of reintroducing the ‘impairment’ criterion to the DSM – we simply point out that its removal has created problems of its own. However, in our opinion, there is no perfect solution to these dilemmas, just compromises. At the end of the day, classifications (DSM, ICD and others) and the way medical departments are structured are rather irrelevant when compared to the demands people in need – *suffering* people – place on us. Calling these people “patients” carries no stigma in our opinion: one wonders why any medical diagnosis should carry stigma, to begin with. Moreover, it can be argued that some bullying and harassment people with TS experience stems precisely from the fact that their tics are not recognized as a movement disorder; rather, they find themselves accused of “doing it on purpose”, of trying to attract attention, etc. We have incorporated these points in the revised version of the manuscript. We have also changed the title by using “condition” instead of “disease”, even though this term cannot be avoided entirely when speaking of research initiatives and networks in this domain. Finally, we would like to remind of the question mark in the title and the ”proposal” in the end: this is an opinion paper supposed to stimulate discussion on an admittedly complicated topic, not scripture.

 As we agree that some people may be incapacitated by their tics and comorbidities, Dr Hedderly indeed points out that our departments are in high demand, with severe caseloads and long waiting lists. And this is where our points of view merge: neither chronic tics nor TS are rare, numerically speaking (again, we simply conform to official definitions of rarity – 1:2000 prevalence rate – which can be debated in itself); but there are not enough trained experts, providers, and infrastructure around to adequately take care of these patients. This, in practical terms, makes TS rare which is, for us, a matter-of-fact statement – but also a call for action. This unsatisfactory situation may be an opportunity to secure support and funding from regional, national, or European bodies to help the TS community – both patients and healthcare providers. One example is COST action BM905 (2010-2014) which was instrumental in creating a European network on TS and gave a tremendous boost to the European Society for the Study of Tourette Syndrome (ESSTS). Since its inception, ESSTS has consistently entertained close ties to patient associations which have now organized in an international network (Tics and Tourette across the Globe - TTAG).

 As to the recent surge in functional tics, these are not the subject of our article, and we do not consider them to be a variant of TS but rather a separate entity (Müller-Vahl et al., 2021). However, we agree that these patients put an additional strain on our already overstretched capacities.

 Concerning definition of evidence-based clusters, this point has also been raised by Dr Nagy, and modifications were made to the manuscript. We agree that this is an important endeavour and, of course, these clusters precisely incorporate comorbidities and move beyond what Dr Hedderly refers to as the ‘tic count’. As TS professionals, we have neither felt nor stated that tics are the sole source of impairment. Likewise, instead of arguing that tics drive social and functional difficulties, we have acknowledged a bidirectional relationship.

 So, to rephrase: TS is not rare but should be supported as such by public bodies, as long as adequate research and medical care is not available in so many countries around the globe. Once TS has become more “mainstream” and features more prominently in the radar of the general public, government agencies, but also the private sector, and across countries and continents, we’ll be prepared to join the ranks of non-rare (common) conditions.

*Müller-Vahl KR, Pisarenko A, Jakubovski E, Fremer C. Stop that! It's not Tourette's but a new type of mass sociogenic illness. Brain. 2021 Aug 23:awab316. doi: 10.1093/brain/awab316. Epub ahead of print. PMID: 34424292.*

# Peer review: Reviewer response for version 1

**DOI:** 10.5256/f1000research.56487.r86525

**Published:** 2021-06-18

**Authors:** Peter Nagy

**Affiliations:** 1 Vadaskert Hospital and Outpatient, Budapest, Hungary

The authors address an important problem Tourette's experts face: by demonstrating how Tourette's syndrome (TS) is not in fact the rare curiosity people had long thought it to be, TS was actually removed from the category of rare diseases, stripping patients of the opportunities rare disease research could bring them. They argue that even though prevalence rates are higher than rare disease definition requirements, the number of patients, especially adult patients with a degree of impairment calling for treatment will be considerably lower, making this subgroup qualify for the rare disease category. 

 The need for intensive research is painfully obvious to experts of the field: with the enormous advances in research technologies over the past decades, it is almost embarrassing to admit that the "exact pathophysiology remains elusive" to this day (Cavanna, 2018), and it is then no surprise that we are far from being able to confidently offer safe and effective treatment options for our patients (the most recent AAN guideline found only moderate confidence for the efficacy of most therapies, except for CBIT, Pringsheim et al., 2019). However, the main argument (TS should be considered a rare disease) needs some careful consideration. It appears from the available data that TS based on its current definition is definitely not a rare disease, so, based on evidence, classifying the diagnosis *per se* as rare will not be possible. Careful and evidence-based definition of subgroups, however, as suggested by the authors, could be a useful route to take, but specific criteria based on evidence will need to be defined. Although for research on certain aspects, like pathogenesis, the rationale for excluding some more frequent presentations of the disorder (e.g. pediatric patients or patients without coprolalia and echolalia) seems difficult to support, the study of other aspects, like pharmacological treatment, will have to be limited to substantially smaller populations, very likely to hit rare disease definitions on one hand, and very unlikely to motivate companies to invest in such research on the other. 

 In conclusion, the accurate definition of evidence-based subgroups could be a promising way forward towards understanding and hopefully better treating this elusive, but very real and sometimes very impairing disorder.

Is the topic of the opinion article discussed accurately in the context of the current literature?

Yes

Are arguments sufficiently supported by evidence from the published literature?

Yes

Are all factual statements correct and adequately supported by citations?

Yes

Are the conclusions drawn balanced and justified on the basis of the presented arguments?

Partly

Reviewer Expertise:

neurodevelopmental disorders, psychopharmacology

I confirm that I have read this submission and believe that I have an appropriate level of expertise to confirm that it is of an acceptable scientific standard.

## References

[1] : The neuropsychiatry of Gilles de la Tourette syndrome: The état de l'art. *Rev Neurol (Paris)* .2018;174(9) : 10.1016/j.neurol.2018.06.006 621-627 10.1016/j.neurol.2018.06.006 30098800

[2] : Comprehensive systematic review summary: Treatment of tics in people with Tourette syndrome and chronic tic disorders. *Neurology* .2019;92(19) : 10.1212/WNL.0000000000007467 907-915 10.1212/WNL.0000000000007467 PMC653713031061209

# Response

**Published:** 2021-07-26

**Authors:** Andreas Hartmann

**Affiliations:** Pitié-Salpêtrière Hospital, Paris, France

We thank Dr Nagy for his thoughtful comments.

 We agree that reclassifying TS is impossible or actually helpful. In the same vein, we have no wish to officially move TS into the rare disease category. However, we wish to alert the public and policy makers that, in real life, TS shares many features with rare diseases, i.e., a certain neglect and underfunding – especially when taking into account its prevalence, which represents a mismatch in our opinion. Therefore, benefitting from certain advantages inherent to rare diseases seems legitimate to us – for the time being. One example given is participating in the European Organization for Rare Diseases (EURORDIS) network, and apply for national or EU funding in this field.

 As to the pharmaceutical industry, it would, as Dr Nagy points out, rather be demotivated to invest into research for TS if it was indeed rare (and again, it isn’t based on prevalence), so that obtaining orphan drug status for TS cannot be considered neither an achievable or desirable aim.

 However, we agree again with Dr Nagy that more research is needed in defining subgroups in TS; given the fluctuation in symptomatology, it would theoretically be quite possible for an individual to be part of different subgroups within her lifetime! Especially adult TS remains very much of black box in terms of prevalence, evolution and long-term prognosis: here, major research efforts (longitudinal and epidemiological) are needed.

# Response

**Published:** 2021-10-04

**Authors:** Andreas Hartmann

**Affiliations:** Pitié-Salpêtrière Hospital, Paris, France

We thank Dr Nagy for his thoughtful comments.

 We agree that reclassifying TS might not be possible or actually helpful. In the same vein, we have no wish to officially move TS into the rare disease category. However, we wish to alert the public and policy makers that, in real life, TS shares many features with rare diseases, i.e., a certain degree of neglect and underfunding – especially when taking into account its prevalence, which represents a mismatch in our opinion. Therefore, benefitting from certain advantages inherent to rare diseases seems legitimate to us – for the time being. One example given is participating in the European Organization for Rare Diseases (EURORDIS) network, and applying for national or EU funding in this field.

 As Dr Nagy points out, the pharmaceutical industry would be rather demotivated to invest into research for TS if it was indeed rare (and again, it isn’t based on prevalence), so that obtaining orphan drug status for TS cannot be considered neither an achievable nor desirable aim.

 However, we agree again with Dr Nagy that more research is needed in defining subgroups in TS; given the fluctuation in symptomatology, it would theoretically be quite possible for an individual to be part of different subgroups within her lifetime! Especially adult TS remains very much of black box in terms of prevalence, evolution, and long-term prognosis: here, major research efforts (both longitudinal and epidemiological) are needed. This point has been expanded and highlighted in the revised version of the manuscript, and two references added.
